# First Cases of SARS-CoV-2 in Iran, 2020: Case Series Report

**DOI:** 10.18502/ijph.v49i8.3903

**Published:** 2020-08

**Authors:** Jila YAVARIAN, Nazanin-Zahra SHAFIEI-JANDAGHI, Kaveh SADEGHI, Somayeh SHATIZADEH MALEKSHAHI, Vahid SALIMI, Ahmad NEJATI, Fatemeh AJA-MINEJAD, Nastaran GHAVVAMI, Fatemeh SAADATMAND, Saeedeh MAHFOUZI, Ghazal FATEMINASAB, Najmeh PARHIZGARI, Akramsadat AHMADI, Kobra RAZAVI, Soad GHABESHI, Mostafa SABERIAN, Elham ZANJANI, Fatemeh NAMAZI, Tayebeh SHAHBAZI, Farshid REZAIE, Hossein ERFANI, Mohammad Mehdi GOUYA, Mohammad NASR DADRAS, Talat MOKHTARI AZAD

**Affiliations:** 1. Department of Virology, School of Public Health, Tehran University of Medical Sciences, Tehran, Iran; 2. Department of Virology, School of Medical Sciences, Tarbiat Modares University, Tehran, Iran; 3. Department of Cellular and Molecular Nutrition, School of Nutritional Sciences and Dietetics, Tehran University of Medical Sciences, Tehran, Iran; 4. School of Allied Medical Sciences, Tehran University of Medical Sciences, Tehran, Iran; 5. Department of Bacteriology, School of Medical Sciences, Tarbiat Modares University, Tehran, Iran; 6. Iranian Center for Communicable Disease Control, Tehran, Iran

**Keywords:** SARS-CoV-2, Iran, Emerging disease, Virus, COVID-19

## Abstract

In Jan 2020, the outbreak of the 2019 novel coronavirus (SARS-CoV-2) in Wuhan, Hubei Province of China spread increasingly to other countries worldwide which WHO declared it as a public health emergency of international concern. Iran was included in the affected countries. Throat swab specimens were collected and tested by using real-time reverse transcription PCR (RT-PCR) kit targeting the E region for screening and RNA dependent RNA polymerase for confirmation. Conventional RT-PCR was conducted for the N region and the PCR products were sequenced by Sanger sequencing. The first seven cases of SARS-CoV-2 infections were identified in Qom, Iran. This report describes the clinical and epidemiological features of the first cases of SARS-CoV-2 confirmed in Iran. Future research should focus on finding the routes of transmission for this virus, including the possibility of transmission from foreign tourists to identify the possible origin of SARS-CoV-2 outbreak in Iran.

## Introduction

From 1960 until 2019, six human coronaviruses (HCoVs) have been recognized which HCoV-229E, OC43, HKU1 and NL63 are known to circulate in the human population, predominantly in young kids (1). The other two HCoVs are severe acute respiratory syndrome coronavirus (SARS-CoV) and Middle East respiratory syndrome coronavirus (MERS-CoV) which are highly pathogenic (2). In Dec 31, 2019, a cluster of patients with lower respiratory infections in people related with the Huanan Seafood Wholesale Market in Wuhan, Hubei Province was reported in China (3, 4). Afterward the seventh HCoV, now named as SARS-CoV-2, was identified in those cases and the disease it causes is called coronavirus disease 2019 (COVID-19). The origin of the new virus was reported to be a bat but now person-to-person transmission of SARS-CoV-2 is occurring (5, 6). After SARS-CoV-2 outbreak in Wuhan, Iranian Ministry of Health has started screening for passengers arriving from China. As of Jul 15, 2020, a total of 13,119,239 confirmed cases had been reported in at least 213 countries with 573,752 deaths (https://www.who.int/emergencies/diseases/novel-coronavirus-2019).

Here we report the first cases of SARS-CoV-2 infections in Qom, central Iran in Feb 2020.

## Materials and Methods

Throat swab specimens were collected and tested by using real-time reverse transcription PCR (RT-PCR) with kits (Modular DxKit, Wuhan CoV E &RdRP genes) provided by WHO targeting the E region for screening and RNA dependent RNA polymerase for confirmation. Conventional RT-PCR was conducted for the N region using N1F and N3R primers reported in CDC protocol (https://www.cdc.gov/coronavirus/2019-ncov/lab/rt-pcr-panel-primer-probes.html). The PCR products of the N region were sequenced by Sanger sequencing method. Alignment and identity matrix analyses were implemented using Multiple Alignment by Multiple Sequence Comparison by Log-Expectation (MUSCLE). MEGA X software was used for construction of Maximum likelihood tree by using the Jukes-Cantor model as chosen by best-fit substitution model.

### The cases

The first patient was a 68-year-old man admitted to hospital A in Qom with severe acute respiratory infection (SARI), chills and dyspnea on Feb 12, 2020. His throat swab sample was collected on Feb 15 and sent to Iran National Influenza Center (NIC) located at School of Public Health, Tehran University of Medical Sciences for influenza screening. His influenza test was negative. His condition deteriorated and on Feb 16, five days after his symptoms began, he died of progressive respiratory failure. His sample was tested for SARS-CoV-2 detection on Feb 18 in NIC and confirmed on Feb 19.

Patient 2 was a 75-year-old man with SARI, chills and dyspnea who became ill on Feb 7, 2020, and admitted to hospital A on Feb 9. After screening his throat swab sample for influenza virus detection in NIC which was negative, he died on Feb 16. His sample was stored in −70 in NIC and after detection of first SARS-CoV-2 on Feb 18, the same test was performed on his sample and reported positive for SARS-CoV-2 on Feb 19.

After detection of the first SARS-CoV-2 cases on Feb 19, immediately the samples were collected from all suspected cases hospitalized in Qom, which 5 of them (5/16) were positive for SARS-CoV-2 (Table 1). They were isolated and treated with supportive therapies and antibiotics.

**Table 1:** Patient’s data on 7 SARS-CoV-2 infections in Qom, Iran, 2020

***Patient No***	***Age(year)/sex***	***Date of symptoms onset***	***Duration of hospitalization***	***Date of infection confirmation***	***Date of death***	***GISAID accession ID***
1	68/M	Feb 7	Feb 12–16	Feb 19	Feb 16	EPI_ISL_414945
2	75/M	Feb 7	Feb 9–16	Feb 19	Feb 16	EPI_ISL_413904
3	68/F	Feb 14	Feb 14–20	Feb 19	Feb 20	EPI_ISL_414373
4	38/F	Feb 16	Feb 16–22	Feb 19	Feb 22	EPI_ISL_414372
5	57/F	Feb 14	Feb 16–20	Feb 19	Feb 20	EPI_ISL_414946
6	55/M	Feb 9	Feb 15–22	Feb 19	Feb 22	EPI_ISL_414374
7	39/F	Feb 10	Feb 16–23	Feb 19	Feb 23	EPI_ISL_414371

Patient 3 was a 68-year-old female. She became ill on Feb 14, 2020, with dyspnea, restlessness and labored breathing and admitted to hospital B on the same day. Her test was positive on Feb 19. Her condition deteriorated and she died on February 20.

Patient 4 was a 38-year-old woman admitted to the hospital C with fever (temperature >38 °C), cough and sore throat on Feb 16, 2020, in two days she was transferred to intensive care unit (ICU) and she died on Feb 22.

Patient 5 was a 57-year-old female with dyspnea, cough, fever (temperature >38 °C) and labored breathing admitted to the hospital A on Feb 16, 2020, her sample was collected on Feb 17 and she passed away on Feb 20.

Patient 6 was 55-year-old man who had fever (temperature >38 °C), anorexia, diarrhea, labored breathing and dyspnea since Feb 9, but admitted to hospital A on Feb 15. His sample was collected on Feb 17 and he died on February 22.

Patient 7 was 39 year old female with fever (temperature >38 °C), dyspnea, anorexia and arthralgia since Feb 10 and hospitalized on Feb 16. She was in hospital A in ICU and died on Feb 23.

**Fig. 1:**
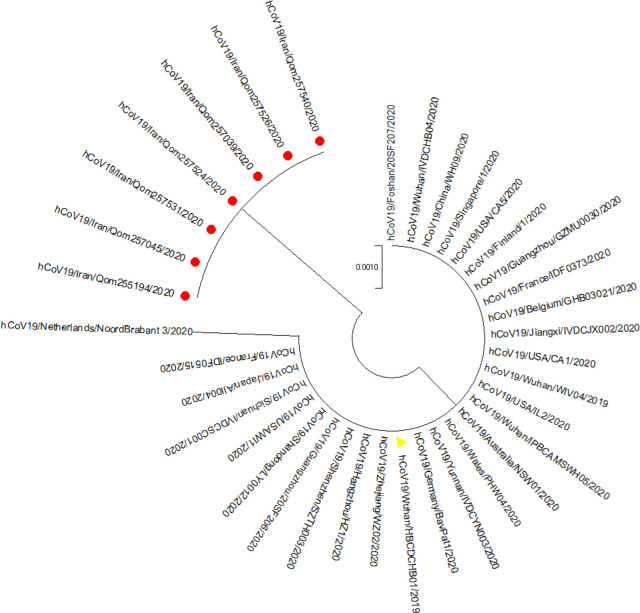
Phylogenic sequence analysis of SARS-CoV-2 N gene detected from patients in Qom, Iran (red circles), 2020, compared with sequences from GSAID. The reference sequence is shown with yellow triangle. MEGA X software was used for construction of Maximum likelihood tree by using the Jukes-Cantor model as chosen by best-fit substitution model

Collectively seven patients’ residents of Qom City were positive for SARS-CoV-2 on Feb 19 in Iran. They were hospitalized in different hospitals with no relation between them. Phylogenetic analysis (Fig. 1) showed some differences between their sequences and a reference sequence retrieved from GISAID (accession ID:EPI-ISL- 402124). All seven sequences from these cases had mutations at position 28688 (T→C). The mutation at position 28688 was nonsynonymous with a leucine to proline change. For all isolates except EPI-ISL-413904, another nonsynonymous mutation was observed at position 28372 (T→G), which resulted in a change of tryptophan to glycine.

## Discussion

This report of the first cases of SARS-CoV2 in Iran showed the evidence of SARS-CoV-2 outbreak in Qom but there are some unclear aspects including the first transmission route. Our two first patients were hospitalized with SARI but their influenza test was negative. Meanwhile all identified cases had signs and symptoms of respiratory infections which were impossible to differentiate clinically from many other viral infections, especially in cold season. Then this report highlights the role of clinicians in proper identification of any suspected cases who might be at risk for any newly identified virus. In this regard after possible diagnosis, the patients can be isolated to reduce virus transmission.

About the epidemiological survey, none of the confirmed cases had history of traveling to China and any contact with suspected patients, visiting health care facilities and live animal markets. Meanwhile foreign tourists from more than 80 countries across the world are visiting the shrine of Hazrat Masoumeh, the daughter of the 7th Shia Imam, in Qom, Iran. Then gathering of millions of visitors might be a possible source of infection in this city.

Lack of enough clinical and laboratory findings, chest radiography, urine, stool and blood specimens of the patients were some of our reports’ limitations.

## Ethical considerations

Ethical issues (Including plagiarism, informed consent, misconduct, data fabrication and/or falsification, double publication and/or submission, redundancy, etc.) have been completely observed by the authors.

## Conclusion

We identified the first cases of SARS-CoV-2 in Qom city, Iran with unclear transmission route. Futureresearch should focus on finding the routes of transmission for this virus, including the possibility of transmission from foreign tourists to identify the possible origin of SARS-CoV-2 outbreak in Iran.

## Ethical approval

Not applicable.

## Funding Sources

This research did not receive any specific grant from funding agencies in the public, commercial, or not-for-profit sectors.

## References

[1] Fielding BC (2011). Human coronavirus NL63: a clinically important virus? Future Microbiol, 6(2): 153–59. 21366416 10.2217/fmb.10.166 PMC7079714

[2] Lim YX Ng YL Tam JP Liu DX (2016). Human coronaviruses: a review of virus–host interactions. Diseases, 4(3): 26. 10.3390/diseases4030026 PMC5456285 28933406

[3] Huang Ch Wang Y Li X (2020). Clinical features of patients infected with 2019 novel coronavirus in Wuhan, China. Lancet, 395 (10223): 497–506. 31986264 10.1016/S0140-6736(20)30183-5 PMC7159299

[4] World Health Organization (2020). Pneumonia of unknown cause – China. https://www.who.int/csr/don/05-january-2020-pneumonia-of-unkown-cause-china/en/

[5] Holshue ML DeBolt C Lindquist S (2020). First case of 2019 novel coronavirus in the United States. N Engl J Med, 382(9): 929–36. 32004427 10.1056/NEJMoa2001191 PMC7092802

[6] Phan LT Nguyen TV Luong QC (2020). Importation and human-to-human transmission of a novel coronavirus in Vietnam. N Engl J Med, 382 (9): 872–74. 31991079 10.1056/NEJMc2001272 PMC7121428

